# Identification of Cultivable Bacteria from Tropical Marine Sponges and Their Biotechnological Potentials

**DOI:** 10.21315/tlsr2018.29.2.13

**Published:** 2018-07-06

**Authors:** Tan Suet May Amelia, Al-Ashraf Abdullah Amirul, Jasnizat Saidin, Kesaven Bhubalan

**Affiliations:** 1 School of Marine and Environmental Sciences, Universiti Malaysia Terengganu, 21030 Kuala Nerus, Terengganu, Malaysia; 2 School of Biological Sciences, Universiti Sains Malaysia, 11800 USM Pulau Pinang, Malaysia; 3 Malaysian Institute of Pharmaceuticals and Nutraceuticals, National Institutes of Biotechnology Malaysia (NIBM), Ministry of Science, Technology and Innovation, 11700 Gelugor, Pulau Pinang, Malaysia; 4 Centre of Chemical Biology, Universiti Sains Malaysia, 11900 Bayan Lepas, Pulau Pinang, Malaysia; 5 Institute of Marine Biotechnology, Universiti Malaysia Terengganu, 21030 Kuala Nerus, Terengganu, Malaysia

**Keywords:** Marine Sponge, Marine Sponge-Associated Bacteria, Biotechnology, South China Sea, Bidong Island, Span Laut, Bakteria Span Laut, Bioteknologi, Laut China Selatan, Pulau Bidong

## Abstract

Marine sponges are acknowledged as bacterial hotspots in the oceanic biome. Aquatic bacteria are being investigated comprehensively for bioactive complexes and secondary metabolites. Cultivable bacteria associated with different species of sea sponges in South China Sea waters adjacent to Bidong Island, Terengganu were identified. Molecular identification was accomplished using 16S rRNA gene cloning and sequencing. Fourteen bacterial species were identified and their phylogenetic relationships were analysed by constructing a neighbour-joining tree with Molecular Evolutionary Genetics Analysis 6. The identified species encompassed four bacterial classes that were Firmicutes, Actinobacteria, Alphaproteobacteria and Gammaproteobacteria known to have been associated with sponges. The potential biotechnological applications of the identified bacteria were compared and reviewed based on relevant past studies. The biotechnological functions of the 14 cultivable isolates have been previously reported, hence reinforcing that bacteria associated with sponges are an abundant resource of scientifically essential compounds. Resilience of psychrotolerant bacteria, *Psychrobacter celer*, in warm tropical waters holds notable prospects for future research.

## Abstract (ms)

Span laut diakui sebagai titik panas bakteria di bioma samudera. Bakteria akuatik sedang diselidiki secara komprehensif untuk kompleks bioaktif dan metabolit sekunder. Bakteria yang boleh dikulturkan dikenal pasti dari spesies span laut berlainan di perairan Laut China Selatan berdekatan Pulau Bidong, Terengganu. Pengenalpastian molekul dicapai dengan pengklonan dan penjujukan gen 16S rRNA. Empat belas spesies bakteria telah dikenalpasti dan hubungan filogenetik mereka telah dianalisis dengan membina pokok *neighbour-joining* menggunakan *Molecular Evolutionary Genetics Analysis* 6. Spesies yang dikenal pasti merangkumi empat kelas bakteria yang pernah dikaitkan dengan span, iaitu kelas Firmikuta, Aktinobakteria, Alfaproteobakteria dan Gammaproteobakteria. Aplikasi bioteknologi bakteria yang dikenal pasti telah dibandingkan dan diulaskan berdasarkan kajian lepas yang berkaitan. Fungsi bioteknologi 14 isolat kultivar telah dilaporkan sebelum ini, justeru meyakinkan bahawa span adalah sumber kaya dengan bahan-bahan yang penting dari segi saintifik. Kewujudan bakteria psikotoleran *Psychrobacter celer* di perairan tropika yang hangat memiliki prospek ketara untuk penyelidikan masa depan.

Progressively known as a wealthy source of significant bioactive compounds, bacteria associated with sea sponges are receiving attention from the scientific community for novel secondary metabolites with desirable economical, pharmaceutical and cosmetic values (Thomas *et al.* 2010). In this study, we identified 14 cultivable bacterial species related with sea sponges from adjacent waters of Bidong Island, Terengganu. Three different marine sponges namely *Xestospongia* sp., *Haliclona* sp. and *Aaptos* sp. were collected and identified. Bacteria associated with these sponges were identified and their biotechnological functions were addressed based on literature and previous researches.

A portion of the sponge surface (approximately 3 cm^2^ × 0.3 cm depth), which included the pinacoderm and mesohyl, was sampled using a sterile scalpel. The sponge tissue was divided into smaller pieces (2 mm^3^) using a sterile scalpel. Each 2 mm^3^ fragment was transferred into an Eppendorf tube. The sponge tissue fragments were washed with 1 mL of 0.22 μm filtered sterile natural seawater by gently shaking for 1 min at room temperature (25 ± 1°C), to remove loosely attached bacteria (Saidin *et al.* 2017). The bacterial isolates went through primary screening based on colonial morphology observation on Zobell marine agar 2216 and Gram (1884) staining. The strains were isolated from the tissues of two *Xestospongia* sp., two *Haliclona* sp., and one *Aaptos* sp. marine sponges. The 16S ribosomal ribonucleic acid (16S rRNA) gene was amplified via direct colony polymerase chain reaction (PCR) by adding magnesium chloride (MgCl_2_), deoxyribonucleotide triphosphate (dNTP) mix, PCR buffer, forward primer 63F (5′-CAGGCCTAACACATGCAAGTC-3′) and reverse primer 1389R (5′-ACGGGCGGTGTGTACAAG-3′), *Taq* polymerase, and sterile distilled water into each tube. A single colony as template was added into each tube using sterilised toothpick (Riviere *et al.* 2013). The Applied Biosystems® Veriti® 96-Well Thermal Cycler (Thermo Fisher Scientific Corp., California, United States) was set with pre-denaturation at 95°C for 5 min, followed by 26 cycles of denaturation, annealing and extension at 95°C for 1 min, 50°C for 30 s, and 70°C for 90 s respectively before proceeding with final extension at 72°C for 5 min (Turner *et al.* 1999).

Subsequently, an agarose gel was prepared by dissolving 0.7% agarose powder in 1x tris-acetate-EDTA (TAE) buffer. The DNA ladder and PCR products with 6X loading dye were loaded into the wells. The loaded gel was run in an electrophoresis machine at a voltage, current and time of 90 V, 35 mA and 75 min respectively using Bio-Rad PowerPac™ Basic Power Supply gel electrophoresis machine (Bio-Rad Laboratories Inc., California, United States) (Sambrook & Russell 2006). The fluorescent Promega Diamond™ Nucleic Acid Dye was used to stain and view the gel using a Gel Doc XR+ Imaging System, which helped to determine the sizes of PCR DNA fragments.

After purification with Promega Wizard® Genomic DNA Purification Kit (Catalog No. A1120, Promega Corp., Wisconsin, United States), the PCR DNA was sequenced at First BASE Laboratories Sdn. Bhd. The DNA sequence for each isolate was identified using Standard Nucleotide BLAST program (BLASTN) to search the nucleotide databases using a nucleotide query, which is one of the programs under the BLAST® (Basic Local Alignment Search Tool, National Library of Medicine, Maryland, USA) program. The sequences were aligned using ClustalW sequence alignment program. A phylogenetic tree of the identified bacteria and their bacterial evolutionary relationships was inferred via Molecular Evolutionary Genetics Analysis version 6 (MEGA6) (Tamura *et al*. 2013).

Fourteen bacteria species were identified by closest similarity with sequence databases using BLAST (Fig. 1). The identified sponge-associated bacteria species were *Alteromonas macleodii*, *Bacillus aquimaris*, *B. aryabhattai*, *B. cereus*, *Brevibacterium epidermidis*, *B. iodinum*, *Enterobacter cloacae*, *Exiguobacterium indicum*, *Kytococcus sedentarius*, *Pseudomonas putida*, *Pseudovibrio denitrificans*, *Psychrobacter celer*, *Ruegeria arenilitoris*, *Staphylococcus hominis* subsp. *novobiosepticus*. The phylogenetic tree in Figure 1 was inferred using the Neighbour-Joining method (Saitou & Nei 1987) with software MEGA6. The percentages of replicate trees where the associated taxa clustered together in the bootstrap test (10,000 replicates) are shown above the branches (Felsenstein 1985). The tree was drawn to scale, with branch lengths in the same units as those of the evolutionary distances used to infer the phylogenetic tree. The evolutionary distances were computed using the Maximum Composite Likelihood method (Tamura *et al.* 2004) and are in the units of the number of base substitutions per site. All positions containing gaps and missing data were eliminated. The phylogenetic tree analysed 15 nucleotide sequences: an ingroup comprising of 14 identified bacteria species and an outgroup of one microalga species.

Fig. 1 shows a phylogenetic tree with the identified bacteria categorised into four bacteria groups, which are the phylum Firmicutes as well as the classes Actinobacteria, Alphaproteobacteria, and Gammaproteobacteria. Bacteria from these similar groups have been previously isolated from marine sponges in geographically disparate areas such as the coastal waters of India, China, Ireland and Brazil (Webster & Taylor 2012). The previously reported geographic and biological isolation sources of these bacteria are shown in Table 1. Majority of species identified in this study were previously isolated from marine sponges, suggesting they were conceivably resident bacteria and not temporarily trapped transient bacteria (Table 1). Out of the 14 identified species, eight were ubiquitously present in more than one ocean and were also isolated from other marine invertebrates, they are *Alteromonas macleodii* (Mehta *et al.* 2014), *Bacillus aquimaris* (Phelan *et al.* 2011, Cerritos *et al*. 2010), *B. aryabhattai* (Pindi *et al.* 2013; Paul *et al.* 2013), *B. cereus* (Maliji *et al.* 2013; Irshad *et al.* 2013), *Enterobacter cloacae* (Maleki-Ravasan *et al.* 2015; Altug *et al.* 2013), *Exiguobacterium indicum* (Jimenez *et al.* 2014; Pindi *et al.* 2013), *Pseudomonas putida* (Altug *et al.* 2013; Irshad *et al*. 2013), and *Pseudovibrio denitrificans* (Santos *et al.* 2014; Rua *et al.* 2014). Ubiquitous bacteria are able to compete against exclusive sponge-associated bacteria for survival in marine hosts due to their flexibility in adapting to diverse environments, namely by exploiting resources, producing secondary metabolites and bioactive compounds, as well as tolerating broad ranges of temperature and salinity (Ivars-Martinez *et al.* 2008). Consequently, the presence of competent ubiquitous bacteria within sponge larvae promotes the survivability of the latter when both symbiont and host are dispersed by ocean current to new habitats lacking in optimum physical parameters or symbionts (Thacker & Freeman 2012). As shown in Table 1, 8 out of 14 identified species were previously reported to have been associated with sponge hosts. A thorough search of relevant literature yielded no previous records that associated sea sponges with the remaining six identified species, which are *Brevibacterium epidermidis*, *Brevibacterium iodinum*, *Ruegeria arenilitoris*, *Kytococcus sedentarius*, *Exiguobacterium indicum*, and *Staphylococcus hominis* subsp. *novobiosepticus*. The presence of human microflora in sponge tissues implied that bacteria from heavily populated proximal waters may have passed through the filter-feeding sponges as temporary transient bacteria during sample collection, since geographically restricted human microflora has low survival rate against competent bacterial associates of sponges. However, future research is needed to study the relationship between the residing bacteria and the indigenous symbiont community, since the genome complexity and metabolic capabilities of the latter may gradually reduce due to exclusively restricted living environments within sponge hosts (Thacker & Freeman 2012).

Table 2 displays the biotechnological applications or potentials recorded for the species recognised in this study. Some were studied for the synthesis of enzymes and pharmaceutical or medical properties (Table 2). Among the identified species, the most common biotechnological function was associated with bioremediation, while subsequent common capabilities were plant growth promotion, plant systemic resistance, and biological syntheses of biocompatible nanometal and biodegradable polymers. We would like to note several interesting species that harbour unique abilities or products, namely lead, cadmium and zinc biosorption mechanism in *A. macleodii* (Loaëc *et al.* 1997); halotolerant and psychrotolerant α-amylase with broad pH tolerance in *A. macleodii* (Han *et al.* 2014); skeletal muscle relaxation properties and central nervous system (CNS) depressants in *B. cereus* (Kumar *et al.* 2014); antileukaemic and antineoplastic L-asparaginase in *B. aryabhattai* (Singh & Srivastava 2014); polyhydroxyalkanoate (PHA) synthesis in *P. putida* (Wang & Nomura 2010); biodegradation of highly explosive pentaerythritol tetranitrate (PETN) by *E. cloacae* (Binks *et al.* 1996); as well as antibacterial, immunosuppressive and anticancer prodigiosin in *P. denitrificans* (Sertan-de Guzman *et al.* 2007).

The commonly recorded capability of bioremediation in some of the identified ubiquitous bacteria is explicable, for the degradation and utilisation of surrounding resource allowed these adaptable bacteria to tolerate severe environments by exploiting nutrients, producing secondary metabolites and bioactive compounds, and tolerating broad ranges of temperature and salinity (Ivars-Martinez *et al.* 2008), hence eventually settling as competent symbionts of marine invertebrates. Moreover, it should be brought to attention that the water bioremediation capacity of *Pseudomonas putida* (Table 2) was not an innate ability but expressed via ice-nucleation protein (INP) surface anchor, which revealed a ten-fold catalytic reaction compared to preceding research on *Escherichia coli* (Shimazu *et al.* 2003). Additional common biotechnological aptitude was the biosynthesis of nanometals that has been earlier reported for two identified species in this study, which are *A. macleodii* and *B. cereus*. Bacteria that biosynthesise nanometal particles are resistant to heavy metals, which could be found in ports and waters polluted with heavy metal effluents (Ramanathan *et al*. 2013).

A lack of previous reports were available for the biotechnological applications of *B. epidermidis*, *R. arenilitoris*, *P. celer*, and *S. hominis* subsp. *novobiosepticus*. Among the four strains, *B. epidermidis* is characteristically found on human skin as an ordinary human microflora. Although research on *B. epidermidis* is deficient, human microflora may harbour unknown dermatological prospects, such as callous treatment discovered in a human microflora recognised as *Kytococcus sedentarius* (Longshaw *et al.* 2002). Furthermore, *P. celer* is of a psychrophilic or psychrotolerant genus, *Psychrobacter* sp., whereby its ability to thrive in both warm and cold regions may be a desirable trait for industrial research purposes (Rodrigues *et al.* 2009). *R. arenilitoris* is a relatively recent species requiring research on its biotechnological prospects (Park & Yoon 2012). Although the recognised species were chiefly ubiquitous bacteria whereby they were neither exclusive nor specific to sea sponges, the results of this research nevertheless correspond with past studies that sponges are abundant with microbes capable of pharmaceutical and biotechnological applications (Schippers *et al.* 2012).

The biotechnological uses shown in Table 2 show ample research prospects among the identified cultivable symbionts associated with sponges. However, we would like to note that the same bacteria species does not necessarily yield similar products due to the mutation or adaptation of strains in respect to their surrounding environment. Majority of the identified species were ubiquitous with proficient survival mechanisms that promote adaptability in harsh environments, thus opportunistically outgrowing adjacent bacteria in marine sponges and residing as a part of the symbiont community in marine sponges. Furthermore, ten identified species were recorded in past studies to have harboured at least two biotechnological purposes, illuminating sponges as a markedly wealthy mine of microbes that are medically and biotechnologically substantial. However, cultivable bacteria under laboratory conditions occupy less than 1% of the total sponge microbial community (Amann *et al.* 1995), suggesting an uncharted existence of valuable research prospects within the putative 99% of uncultivated sponge bacteria. As a result, research is essential on growth media that foster the development of sponge symbionts, and on progressive metagenomic approaches that access the environmental metagenome devoid of isolation on complex culture media.

## Figures and Tables

**Figure 1:**
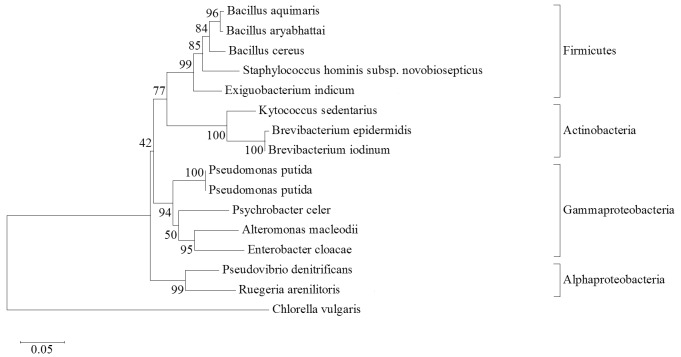
Phylogenetic tree with 14 species ingroup and one microalga outgroup.

**Table 1:** Sponge hosts and geographic locations of the isolated bacteria species.

Isolated species	Sponge hosts	Geographic locations	References
*Alteromonas macleodii*	*Callyspongia sp.*	Hong Kong	Qian *et al.* (2006)
	*Gelliodes carnosa*	Xincun Harbour, Hainan	Li *et al.* (2011)
*Bacillus aquimaris*	*Haliclona simulans*	Galway, Ireland	Phelan *et al.* (2011)
	*Gelliodes carnosa*	Xincun Harbour, Hainan	Li *et al*. (2011)
*Bacillus aryabhattai*	*Sarcotragus fasciculatus*	Bay of Bengal, India	Pandey *et al.* (2013)
*Bacillus cereus*	*Haliclona simulans*	Galway, Ireland	Phelan *et al.* (2011)
	*Callyspongia sp.*	Hong Kong	Qian *et al.* (2006)
	*Hyattella cribriformis*	India	Brammavidhya & Usharani (2013)
	*Sigmadocia sp.*	Kanyakumari coast	Satheesh *et al.* (2012)
	*Spirastrella abata*	Moseulpo Port, Jeju Island	Cho & Park (2009)
	*Spirastrella panis*	Moseulpo Port, Jeju Island	Cho & Park (2009)
	*Polymastia janeirensis*	Rio de Janeiro	Santos-Gandelman *et al.* (2014)
	*Arenosclera brasiliensis*	Rio de Janeiro	Rua *et al*. (2014)
	*Stelletta tenui*	Sanya, Hainan	Li *et al*. (2007)
	*Dysidea avara*	Sanya, Hainan	Li *et al*. (2007)
	*Gelliodes carnosa*	Xincun Harbour, Hainan	Li *et al.* (2011)
*Enterobacter cloacae*	*Dysidea granulosa*	Kavaratti Islands, India	Gopi *et al.* (2012)
*Pseudomonas putida*	*Mycale microsigmatosa*	Rio de Janeiro	Marinho *et al.* (2009)
*Pseudovibrio denitrificans*	*Callyspongia plicifera*	Bahamas	Qian *et al.* (2006)
	*Spirastrella panis*	Moseulpo Port, Jeju Island	Cho & Park (2009)
	*Spirastrella abata*	Moseulpo Port, Jeju Island	Cho & Park (2009)
	*Arenosclera brasiliensis*	Rio de Janeiro	Rua *et al.* (2014)
	*Mycale microsigmatos*	Rio de Janeiro	Santos *et al.* (2014)
*Psychrobacter celer*	*Spirastrella abata*	Moseulpo Port, Jeju Island	Cho & Park (2009)

**Table 2:** Review of potential biotechnological applications of the isolated bacteria species.

Isolated species	Biotechnological application	References
*Alteromonas macleodii*	Metallic ion biosorption	Loaëc *et al.* (1997)
	Hydrogenase enzyme production	Vargas *et al.* (2011)
	α-amylase enzyme production	Han *et al.* (2014)
	Biocompatible nanometal production	Mehta *et al.* (2014)
	Alkaline phosphatase production	Fedosov *et al.* (1991)
*Bacillus aquimaris*	Alkaline cellulase production	Trivedi *et al.* (2011)
	α-amylase enzyme production	Puspasari *et al.* (2013)
*Bacillus aryabhattai*	β-glucosidase inhibitor production	Pandey *et al.* (2013)
	L-asparaginase enzyme production	Singh & Srivastava (2014)
	Polluted water bioremediation	Chen *et al.* (2012)
	Bagasse degradation	Wen *et al.* (2015)
*Bacillus cereus*	Antimicrobial	Brammavidhya & Usharani (2013)
	Therapeutic depressant potential	Kumar *et al.* (2014)
	Petroleum hydrocarbon degradation	Maliji *et al.* (2013)
	Mercury bioremediation	Santos-Gandelman *et al.* (2014)
	Silver nanoparticle production	Babu *et al.* (2011)
*Brevibacterium iodinum*	Plant germination and growth promotion	Kim *et al.* (2012)
	Iodinin antibiotic production	Podojil & Gerber (1967)
*Enterobacter cloacae*	Greenhouse cucumber growth regulator	Georgieva (2003)
	Explosives biodegradation	Binks *et al*. (1996)
*Exiguobacterium indicum*	Wastewater bioaugmentation	Iyer & Iken (2013)
	Manganese mobilisation	Sujith *et al*. (2014)
*Kytococcus sedentarius*	Keratin-degrading enzyme production	Longshaw *et al.* (2002)
	Monensin antibiotic production	Pospisil *et al*. (1998)
*Pseudomonas putida*	Antimicrobial	Marinho *et al*. (2009)
	Polyhydroxyalkanoates (PHA) production	Wang & Nomura (2010)
	Plant systemic resistance induction	Planchamp *et al.* (2015)
	Polluted water bioremediation	Shimazu *et al*. (2003)
	Certified host-vector biosafety strain (HV1)	Federal Register (1982)
*Pseudovibrio denitrificans*	Pseudovibrocin peptide production	Vizcaino *et al*. (2010)
	Prodigiosin pigment production	Sertan-de Guzman *et al*. (2007)
